# Health Behaviours among Travellers Regarding Risk Compensation Following COVID-19 Vaccination in Taizhou, China

**DOI:** 10.1155/2023/1329291

**Published:** 2023-02-25

**Authors:** Meng-Ge Yang, Li-Jun Wang, Lu-Yin Xu, Mang Ke, Liang-Xue Sun

**Affiliations:** ^1^Department of Emergency, Taizhou Hospital of Zhejiang Province Affiliated to Wenzhou Medical University, Enze Hospital, Taizhou Enze Medical Center (Group), Taizhou, Zhejiang, China; ^2^Department of Urology, Taizhou Hospital of Zhejiang Province Affiliated to Wenzhou Medical University, Enze Hospital, Taizhou Enze Medical Center (Group), Taizhou, Zhejiang, China; ^3^Department of Urology, Taizhou Hospital of Zhejiang Province Affiliated to Wenzhou Medical University, Linhai, Zhejiang, China

## Abstract

**Background:**

During the COVID-19 pandemic, public transport was restricted in many countries because of the transmission risk. According to the risk compensation theory, travellers post-COVID-19 vaccination may encounter higher risks; however, no real-world studies provide such evidence. Therefore, we conducted a survey to assess whether risk compensation would occur among travellers' health-related behaviours after COVID-19 vaccination, potentially aggravating the transmission of the virus.

**Materials and Methods:**

A self-administered online survey was designed and distributed over WeChat to identify the difference in health behaviours before and after COVID-19 vaccination among travellers at a train station in Taizhou, China, from 13 February to 26 April 2022.

**Results:**

A total of 602 individuals completed the questionnaire. The results revealed no statistical difference between the health behaviours reported by the vaccinated and unvaccinated groups. Participants who received the first dose of the vaccine earlier showed no statistical difference in harmful health behaviours (hand washing frequency decreased by 4.1% (*P*=0.145) and the duration of public transport travel increased by 3.4% (*P*=0.437)), but showed better protective health behaviours (mask-wearing duration increased by 24.7% (*P*=0.014)). Compared to those vaccinated less than three times, participants vaccinated against COVID-19 three times showed no statistical differences in harmful health behaviours mask-wearing duration decreased by 7.0% (*P*=0.927), their hand washing frequency decreased by 4.8% (*P*=0.905), and the duration of public transport travel increased by 2.5% (*P*=0.287). After vaccination, when compared to themselves before vaccination, participants exhibited better health behaviours (increased hand washing frequency and mask-wearing duration, and decreased duration of public transport travel) to some extent.

**Conclusion:**

In conclusion, this study found no evidence of risk compensation among travellers. After being vaccinated, health behaviours partly improved among travellers.

## 1. Introduction

COVID-19 is caused by the severe acute respiratory syndrome coronavirus 2 (SARS-CoV-2) and first occurred in Wuhan, Hubei Province, China. It was declared a pandemic by the World Health Organization on January 30, 2020 [1]. More than 287 million cases had been reported globally by December 31, 2021, with over 5.4 million deaths [2]. Due to its highly infectious nature, many countries have restricted public transport in areas where COVID-19 could be easily transmitted among moving people in transit and enclosed spaces [3]. A study in China reported that travelling by public transport (buses and metro) decreased dramatically during the COVID-19 outbreak [4].

Unfortunately, few of the available antiviral treatments can be used to combat COVID-19 [5]. However, individuals naturally adjust their behaviour to minimise risk in precarious situations [6]. Initially, individuals who considered COVID-19 to be threatening undertook safety measures such as washing hands, wearing masks, avoiding large crowds, and maintaining a social distance when cases began to surge [7]. Moreover, wearing face masks in public places to reduce the risk of infection was recommended or mandated in over 160 countries during the COVID-19 pandemic [8, 9].

However, safety measures may have weakened following the development of the COVID-19 vaccine, which is regarded as the panacea to the pandemic [6]. The risk compensation theory hypothesizes that individuals may exhibit riskier behaviour in situations where they feel safer and would take prior responsibility for the phenomenon [10].

This phenomenon, also known as the “Peltzman Effect,” was first described by University of Chicago economist Sam Peltzman in 1975 [6]. Evidence of a relationship between vaccines and risk compensation theory exists: for instance, the use of condoms decreased among women after the administration of a potential human immunodeficiency virus (HIV) vaccine [11], and young females tend to partake in risky sexual behaviours after human papillomavirus (HPV) vaccinations [12]. Thus, in combating the pandemic, the protective effect brought by the COVID-19 vaccine may be reduced due to risk compensation behaviours exhibited postimmunisation [10].

According to the risk compensation theory [13], individuals undertaking protective interventions are likely to exhibit increased harmful behaviours and be exposed to higher risk. Thus, regarding the COVID-19 vaccine, it can be assumed that vaccinated individuals may abandon wearing face masks and washing hands and meeting more people, thus exposing themselves to higher-risk situations [14]. Prior to the current study, we had conducted a questionnaire survey among healthcare workers in a tertiary hospital to address the hypothesis and found no evidence of risk compensation [15]; nonetheless, some real-world evidence supports this hypothesis [6, 16, 17].

In this study, we conducted a survey to assess whether risk compensation would occur among travellers' health-related behaviours after COVID-19 vaccination, potentially aggravating the transmission of the virus.

## 2. Materials and Methods

### 2.1. Study Design and Population

**Figure 1:**
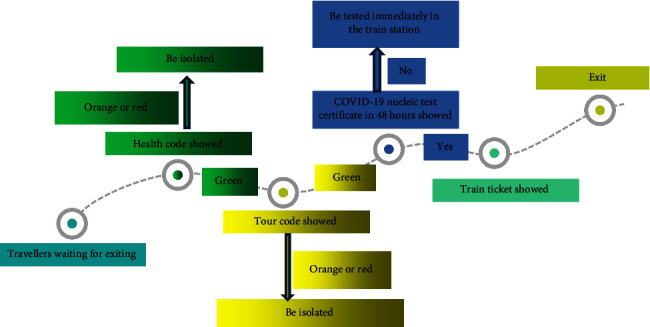
Flowchart for travellers exiting from the train station.

**Figure 2:**
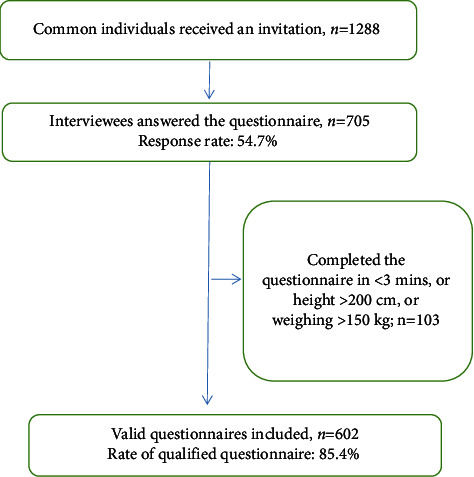
Flowchart of sample size determination method.

The survey was conducted anonymously and without charge to users of the Wen-Juan-Xing platform incorporated into WeChat (Changsha Ranxing Information Technology Co., Ltd., Hunan, China), one of the largest online survey platforms in China [15]. The target population consisted of individuals devoid of any primary COVID-19 symptoms, such as fever, cough, or asthenia, who were exiting Taizhou train station in Taizhou, China. The method for exiting the Taizhou train station during the COVID-19 pandemic is shown in Figure 1. Inclusion criteria stipulated that the participants be familiar with comprehending and reading Chinese and using mobile phone software. We excluded participants with heights >200 cm or weighing >150 kg (based on the data from local government's demographic database, these anthropomorphic characteristics were treated as an extremely rare baseline characteristics for the local population) or who had completed the questionnaire too quickly (<3 mins; completion in this time was determined as to not guarantee careful answers based on number of questions in the questionnaire and self-testing quality before issuing the questionnaire) as ineligible.  Figure 2 shows that 1,288 travellers received a questionnaire for reporting their health behaviours during the COVID-19 pandemic through WeChat. Of these, 705 respondents (response rate, 54.7%) voluntarily answered the questionnaire between 13 February and 26 April 2022. From a total of 705 respondents, 103 travellers met the exclusion criteria. We received 602 questionnaires containing valid data (qualified questionnaire rate, 85.4%). We recorded respondents' age, height, gender, knowledge, occupation, and health behaviours such as duration of travelling by public transport and mask-wearing behaviour (including the type of mask: disposable surgical masks, N95 masks, and nonmedical masks). In addition, information regarding negative emotions experienced (fear, anxiety, and panic) during the COVID-19 pandemic was collected. Harmful health behaviours included decreased hand washing frequency, increased duration of public transport travel, and decreased mask wearing duration. Protective health behaviours included increased hand washing frequency, decreased duration of public transport travel, and increased mask-wearing duration. Participants reported health behaviours before and after receiving the COVID-19 vaccination separately. Data for all individuals who took part in this survey were anonymised. The vaccine referred to in this study was CoronaVac, which uses an inactive form of SARS-CoV-2 and is manufactured by Sinovac Life Sciences (Beijing, China). All participants were vaccinated at no charge as per the Chinese government initiative. The Ethics Committee of Enze Hospital in Taizhou, China, approved this survey (approval number: K20210501), and informed consent was obtained from all participants.

### 2.2. Questionnaires

The questionnaire was used to note the following aspects:

1. General demographic information: height, weight, age, gender, occupation, and education.
2. Health behaviours; assessed using two questions, the first concerning the use of public transport and the second inquiring about the use and type of masks.
3. COVID-19 vaccination status of the participants in this survey was assessed using the following questions: “Have you received the COVID-19 vaccine?” “When were you first vaccinated, if you were,” and “If you were, how many times?”
4. Among vaccinated participants: travelling duration by public transport, mask-wearing duration, and hand washing frequency when travelling among vaccinated participants before and after the COVID-19 vaccination.
5. Negative emotions (anxiety, fear, and panic) experienced during the COVID-19 pandemic.

### 2.3. Statistical Analysis

We analysed the collected data using IBM SPSS Statistics 25.0 software (IBM Inc., Chicago, IL, USA). The univariate analysis assessed categorical variables using *χ*^2^-test in the model. A *P* value of <0.05 was considered statistically significant.

## 3. Results

### 3.1. Study Population Characteristics

In total, 602 travellers in Taizhou, China, comprising 150 men (24.9%) and 452 women (75.1%), completed the questionnaire. All participants answered the survey questions about health behaviours, vaccination status, and negative emotions during the COVID-19 pandemic. Of all the participants, 579 (96.2%) received at least one dose of the COVID-19 vaccination, and 23 (3.8%) were unvaccinated. One participant reported never wearing a face mask during the pandemic. The demographic variables of participants' height, weight, age, gender, occupation, and education level are provided in Table 1.

### 3.2. Health Behaviours of Travellers

In this study, we compared the health behaviours of vaccinated and unvaccinated participants. Tables 2 and 3 show that health behaviours were not associated with vaccination.

### 3.3. Differences in Health Behaviours among Vaccinated Travellers

**Figure 3:**
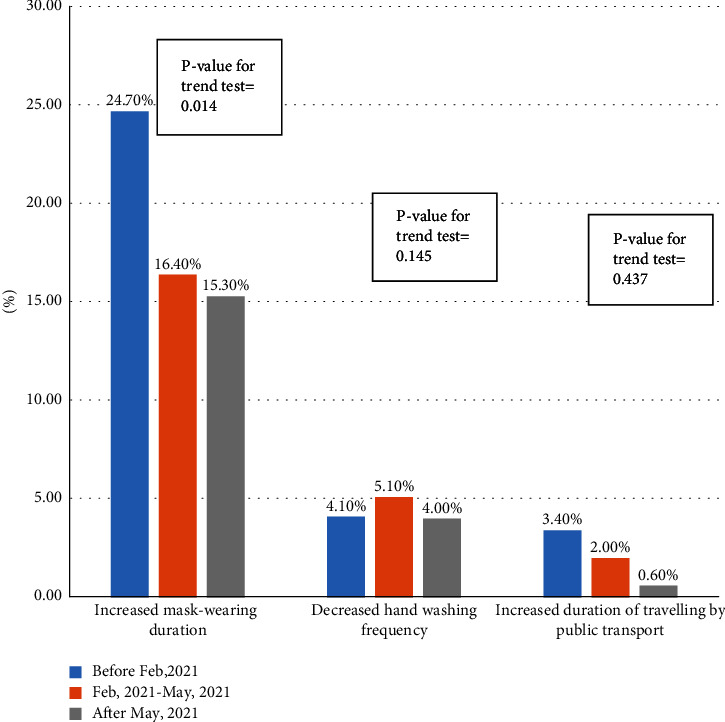
Association between time of first dose of vaccination received and health behaviours.

**Figure 4:**
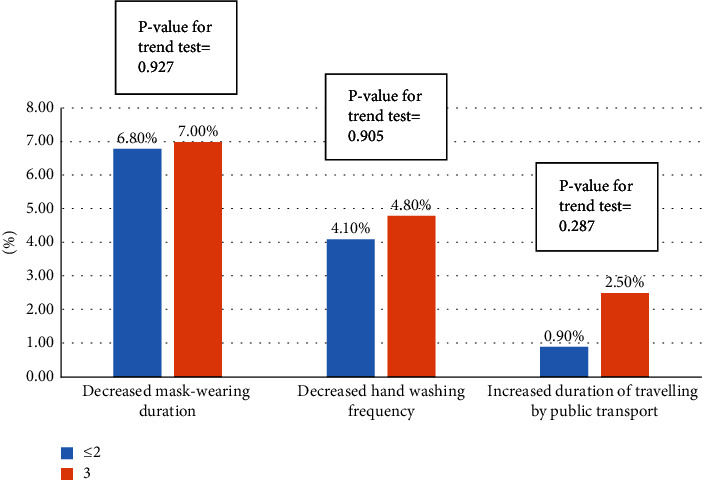
Association between number of vaccine doses received and harmful health behaviours.

**Figure 5:**
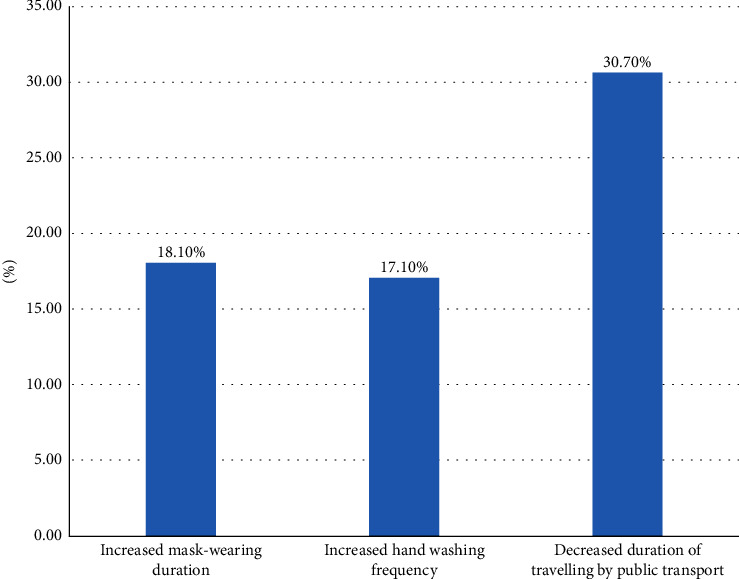
Comparison of travellers' health behaviours before and after vaccination.

Based on the date of the first COVID-19 vaccine dose received, the duration of mask-wearing when travelling participants who received their vaccination earlier increased more unexpectedly (*P*=0.014); in contrast, the frequency of hand washing and the duration of travelling by public transport showed no statistically significant difference (Figure 3). Regardless of the number of doses of the COVID-19 vaccine received, the duration of mask-wearing and of travelling by public transport and the frequency of hand washing did not show statistically significant differences (Figure 4). However, participants exhibited better health behaviours after vaccination (increased hand washing frequency, increased mask-wearing duration, and decreased duration of public transport travel) to some extent when compared to themselves before vaccination (Figure 5).

### 3.4. Experience of Negative Emotions (Fear, Anxiety, and Panic)

Our results show that 54.7% of respondents reported that they had never experienced negative emotions during the COVID-19 pandemic. Among the participants who reported having experienced some degree of negative emotions during the pandemic, a 48% reduction in negative emotions experienced was reported by respondents after vaccination (Table 4).

## 4. Discussion

### 4.1. Clinical Implications

To date, this is the first study to investigate the relationship between COVID-19 vaccination status and health behaviours among travellers. Our study provides real-world data as evidence of this relationship.

The risk compensation theory regards four main factors as likely contributors to understanding risk visibility, motivation, control, and effectiveness, all of which were present during the COVID-19 pandemic [6]. According to the risk compensation theory, individuals may abandon wearing masks, washing hands, and maintaining social distance if they are aware that others have received the vaccination, regardless of their own vaccination status [6].

Owing to its importance for intervention design in practice, the risk compensation theory has gained attention among researchers in diverse areas. These include motor vehicle regulations (for improving occupant and nonoccupant safety) [18], helmet regulations (for improving bicycle rider safety) [19], human papillomavirus vaccination (for preventing cervical cancer) [20], and preexposure prophylaxis (for preventing HIV infections) [21]. An exploratory qualitative study among young South Africans (aged 18 to 24 years) exploring perceptions of risk compensation with regard to HIV vaccination revealed reductions in condom use, an increase in multiple partners, and an increased frequency of intercourse. The study suggested that concerns about the impact of possible risk compensation in HIV vaccine-targeted populations should be incorporated into strategies for vaccine introduction [22]. In addition, a different study noted that 10% of participants would decrease condom use after receiving HIV vaccination [11].

Since the emergence of the COVID-19 pandemic, some studies support risk compensation with interventions related to COVID-19. A report by Trogen and Caplan revealed how risk compensation relates to the COVID-19 pandemic by observing “pandemic fatigue” and postvaccination behaviour [6]. In a study by Yan et al., it was found that on facing mask orders, Americans reduced their time at home by 11–24 minutes on average and increased visits to some high-risk places [16]. The study suggested that face mask orders lead to risk compensation behaviours, as further supported in a paper by Wadud et al. [17], but this was contradicted by Liebst et al. [9]. Another paper investigating the relationship between the COVID-19 vaccine and risk compensation noted that risk compensation could significantly reduce the protection provided by the COVID-19 vaccine, particularly with less efficacious vaccines used [10].

Our study found that travellers do not incorporate more protective interventions, including wearing masks and washing hands when travelling, or more harmful behaviours, such as travelling by public transport after receiving the COVID-19 vaccination. The above finding is also supported by the reports of Thorpe et al. [23], Hall et al. [24], and Guenther et al. [25], who also investigated the risk compensation behaviour during the COVID-19 pandemic. The reason for this finding may be scepticism among participants regarding the vaccine and the advertised high infectivity of COVID-19 in China [1]. In addition, our study indicates that participants who received the vaccination early increased their mask-wearing duration. Furthermore, no apparent improvements were found in the frequency of hand washing and duration of travel by public transport.

More than half of the participants reported they experienced no negative emotions during the COVID-19 pandemic, which was contrary to the reports of Wang et al. and Li et al. [26, 27]. The reasons for this may be as follows: first, our study was completed from 13 February to 26 April 2022, while the epidemic situation in China has been predominantly under control since 8 April 2020 [28]. Secondly, most participants in our study received the COVID-19 vaccination. Thirdly, the government improved the transparency of information by providing daily updates on the epidemic, which improved residents' confidence [28].

However, large-scale and multicentre studies should be conducted to further investigate whether actual risk compensation behaviours following COVID-19 vaccination occur in other real-world scenarios.

### 4.2. Methodological Considerations

We hereby note the limitations of the present study. First, self-administered online questionnaires are prone to yield inaccurate information. However, a data check was executed, and any nonlogical data (data that met the exclusion criteria was defined as nonlogical) was not included in efforts to minimise inaccuracies. Secondly, as the respondents completed the questionnaire by recalling their behaviours, inaccurate information may have been introduced. Additionally, the response rate in the study was relatively low, and the health behaviours of travellers may be affected by China's strict control measures and the current prevalence of COVID-19. However, except in some regions, the epidemic situation in China has been predominantly under control since 8 April 2020, with outbreaks occurring on a small scale [28]. In the stable stage (from 08 April 2020 to the present), the relaxed social blockade and economic recovery had become the core policy adopted by the Chinese government [28], with the overall prevalence and control policy of COVID-19 in China in recent years being mostly stable. Moreover, the limited sample size in the unvaccinated group may be underrepresented. Thus, further large-scale, real-world studies are necessary to investigate whether the concept of risk compensation would prevail among people's health behaviours after COVID-19 vaccination. Lastly, because of the inclusion criteria concerning the use of mobile phone software, it is likely that the respondents were younger, and thus possibly healthier than the general population; hence, selection bias may have occurred.

## 5. Conclusions

The present study found no evidence of risk compensation among travellers. Moreover, there was no decrease in protective health behaviours nor any increase in harmful health behaviours after receiving the COVID-19 vaccine; interestingly, there was an improvement in some health behaviours among travellers.

## Figures and Tables

**Table 1:** Baseline characteristics of the participants.

	Variables	All (*n* = 602) (%)	Vaccinated (*n* = 579) (%)	Unvaccinated (*n* = 23) (%)	*χ* ^2^ or *T*	*P* value
Gender	Men	150 (24.9)	143 (24.7)	7 (30.4)	0.402	0.526
	Women	452 (75.1)	436 (75.3)	16 (69.6)		

Height	(cm)	163.52 ± 6.868	163.50 ± 6.778	164.13 ± 9.017	−0.432	0.666
Weight	(kg)	60.05 ± 13.846	60.01 ± 13.871	61 ± 13.46	−0.336	0.737
Age	(*y*)	31.75 ± 10.98	31.67 ± 11.007	33.81 ± 10.384	−0.918	0.359

Education	Junior college and below	362 (60.1)	349 (60.3)	13 (56.5)	0.130	0.718
	Undergraduate and above	240 (39.9)	230 (39.7)	10 (43.5)		

Occupation					1.926	0.165
	Specialized persons (including teachers, lawyers and doctors)	131 (21.8)	130 (22.5)	1 (4.3)		
	Institutional persons (including civil servants and governmental employees)	102 (16.9)	100 (17.3)	2 (8.7)		
	Students	107 (17.8)	101 (17.4)	6 (26.1)		
	Others	262 (43.5)	248 (42.8)	14 (60.9)		

**Table 2:** Participant behaviours during the COVID-19 pandemic.

Variables	All (*n* = 602) (%)	Vaccinated (*n* = 570) (%)	Unvaccinated (*n* = 23) (%)	*χ* ^2^	*P*
Always travelling by public transport before				0.156	0.693
Yes	364 (60.5)	351 (60.6)	13 (56.6)		
No	238 (39.5)	228 (39.4)	10 (43.5)		
Always wear masks when travelling				0.746	0.388
Yes	366 (60.8)	354 (61.1)	12 (52.2)		
No	236 (39.2)	225 (38.9)	11 (47.8)		

**Table 3:** Behaviours of participants who wore a face mask during the COVID-19 pandemic.

Variables	All (*n* = 601) (%)	Vaccinated (*n* = 578) (%)	Unvaccinated (*n* = 23) (%)	*χ* ^2^	*P*
Source of face masks				0.032	0.858
Bought	434 (72.2)	417 (72.1)	17 (73.9)		
Donated by others	28 (4.7)	27 (4.7)	1 (4.3)		
Reserved	139 (23.1)	134 (23.2)	5 (21.7)		
Type of masks				2.471	0.116
Surgical masks	482 (80.2)	467 (80.8)	15 (65.2)		
Nonmedical/N95 masks	119 (19.8)	111 (19.2)	8 (34.8)		
Time of changing masks				0.157	0.692
<6 h	336 (55.9)	325 (56.2)	11 (47.8)		
6 h–12 h	221 (36.8)	210 (36.3)	11 (47.8)		
>12 h	44 (7.3)	43 (7.4)	1 (4.3)		
Squeeze the tip of mask when wearing masks				1.896	0.169
No	334 (55.6)	318 (55.0)	16 (69.6)		
Yes	267 (44.4)	260 (45.0)	7 (30.4)		
Mask nose and mouth				0.331	0.565
No	227 (37.8)	217 (37.5)	10 (43.5)		
Yes	374 (62.2)	361 (60.1)	13 (56.5)		
Always washing hands				0.160	0.689
No	368 (61.2)	353 (61.1)	15 (65.2)		
Yes	233 (38.8)	225 (38.9)	8 (34.8)		

**Table 4:** Experience of negative emotions (anxiety, panic, and fear) related to COVID-19 prevalence and vaccine inoculation in participants.

Experience of negative emotions during COVID-19 prevalence	
	All (*n* = 602)%
Always	7 (1.2)
Usually	20 (3.3)
Seldom	246 (40.9)
Never	329 (54.7)
Experience of negative emotions postinoculation compared to preinoculation in participants of the COVID-19 vaccine	
	All (*n* = 273)%
Increased	13 (4.8)
Equally	129 (47.3)
Decreased	131 (48)

## Data Availability

The data supporting the findings of this study are available from the corresponding author upon reasonable request.
